# A different approach to cystinosis: ultrasound, doppler, and shear wave elastography findings of thyroid gland

**DOI:** 10.1186/s13023-023-02783-6

**Published:** 2023-06-30

**Authors:** Derya Bako, Sebile Kılavuz, Adem Yasin Köksoy, Zeynep Uzan Tatli, Engin Beydogan

**Affiliations:** 1 Department of Pediatric Radiology, Van Regional Training and Research Hospital, Van, Turkey; 2 Department of Pediatric Pediatric Metabolism and Nutrition, Van Regional Training and Research Hospital, Van, Turkey; 3 Department of Pediatric Pediatric Nephrology, Van Regional Training and Research Hospital, Van, Turkey; 4 Department of Pediatric Pediatric Endocrinology, Zeynep Uzan Tatli, Van Regional Training and Research Hospital, Van, Turkey; 5 Department of Radiology, Van Regional Training and Research Hospital, Van, Turkey

**Keywords:** Cystinosis, Doppler, Elastography, Stiffness, Thyroid, Ultrasound

## Abstract

**Background:**

While thyroid dysfunction develops in about 50% of untreated children with cystinosis, there is no data about how the sonography of thyroid tissue appears in this disease. Therefore, the purpose of this study was to assess the sonographic appearance, color doppler findings in this disease and to evaluate how cystine crystal accumulation affect tissue stiffness using shear wave elastography (SWE).

**Methods:**

Sixteen children diagnosed with cystinosis and a control group consisting of 34 healthy children were included in this study. B mode ultrasound, color doppler imaging and real-time SWE of thyroid tissue were performed.

**Results:**

Ultrasound imaging revealed lower echogenicity and diffuse heterogeneous echotexture in 7 of the 16 cystinosis patients. Thyroid gland volumes were lower in cystinosis patients (p 0.005). Doppler ultrasound demonstrated increased flow in 8 patients. On SWE, the thyroid tissue stiffness was established to be lower in patients compared to healthy children (p 0.003).

**Conclusions:**

This is the first study evaluating thyroid gland B mode, color doppler ultrasonography, and SWE findings in cystinosis. Our findings indicate that cysteamine treatment still cannot completely prevent the disease infiltration process of thyroid gland. The other important finding—that thyroid tissue stiffness was established to be lower than that of the controls—also demonstrates the ongoing disease infiltration process.

## Background

Nephropathic cystinosis is a rare autosomal recessive lysosomal storage disease caused by defective lysosomal membrane transport, encountered in approximately 1 in 100,000 to 200,000 live births [1]. Mutations of the CTNS gene, which is located on chromosome 17p13, are responsible for intracellular accumulation of cystine in various organs and tissues [2]. Although patients usually do not have any distinct laboratory findings at birth, they gradually develop renal tubular Fanconi syndrome by the age of 6–12 months. If not treated, patients develop kidney insufficiency within the first decade and require dialysis or kidney transplantation for survival [3]. While renal Fanconi syndrome is curable through transplantation, other system involvements continue, causing hypothyroidism, diabetes mellitus, hypogonadism, retinal blindness, decreased pulmonary function, myopathies, central nervous system involvement, severe growth retardation and rachitis [1, 4, 5].

Since 1987, the therapy of cystinosis has been centred on oral cysteamine treatment, which was approved by the FDA in 1994 [6]. Early diagnosis and effective treatment with cysteamine decrease intracellular cystine content and delay deterioration of renal function and severity of other extrarenal complications [5, 7–9]. In recent decades, when renal involvement has been slowed down by the treatment and increased longevity of patients, more attention has been focused on extrarenal complications.

Thyroid dysfunction is the most common and earliest endocrine disorder observed in patients with cystinosis, affecting approximately 50% of untreated children. Typically, thyroid dysfunction manifests after kidney dysfunction and becomes clinically apparent between the ages of 5 and 10 [1]. Although cysteamine treatment generally prevents hypothyroidism in the majority of cases, the presence of pituitary resistance to thyroxine may introduce complexity to the progression of thyroid dysfunction [10].” Thyroid gland sonography, which is a touchstone in the evaluation of other diseases involving thyroid tissue, it has never been used in cystinosis. Therefore, the purposes of this study were to assess thyroid gland appearance, echo texture and size on B mode ultrasonography, to evaluate doppler ultrasonography findings and to determine how cystine crystal accumulation and disease progression affect tissue stiffness in these patients, using the recently introduced imaging method of shear wave elastography (SWE).

## Materials and methods

### Patients group

Children diagnosed with infantile nephropathic cystinosis who were followed up at Paediatric Metabolism, Nephrology and Endocrinology Departments of our hospital were included in study.

All patients had symptoms of renal Fanconi syndrome during the diagnosis including polyuria, electrolyte imbalances, glucosuria, phosphaturia, generalized proximal tubular dysfunction, and growth failure. The diagnosis of cystinosis was confirmed by the presence of corneal cystine crystals on eye examination and/or elevated leukocyte cystine levels (> 2 nmol half-cystine per mg protein) and/or CTNS gene mutation(s).

To assess kidney function of cystinosis patients; serum creatinine levels were measured using an enzymatic method. The estimated glomerular filtration rate (eGFR) was calculated using the bedside Schwartz GFR equation, which considers factors such as height (L) and serum creatinine (PCR) [11]. The equation used is as follows: eGFR = k × L (cm) / PCR (mg/dL), where k is 0.33 for a preterm infant, 0.45 for a full-term infant, 0.55 for children and female adolescents, and 0.70 for male adolescents.

Since all cases had structural or functional abnormalities of the kidney persisting for at least 3 months, chronic kidney disease (CKD) was staged based on the level of GFR according to the Kidney Disease Improving Global Outcome (KDIGO) guidelines (KDIGO 2012 Clinical Practice). The CKD stages based on eGFR values were presented as follows: G1 (≥ 90) indicating normal or high, G2 (60–89) indicating mildly decreased, G3a (45–59) indicating mild to moderately decreased, G3b (30–44) indicating moderate to severely decreased, G4 (15–29) indicating severely decreased, and G5 (< 15) indicating kidney failure. CKD stage,

In all cystinosis cases, TSH, T3, and T4 levels were measured prior to ultrasound examination. Autoimmune thyroiditis was ruled out in hypothyroid cases through the assessment of anti-thyroid antibodies.

### Control group

The control group consisted of 34 healthy children. Healthy children were recruited from patients referred for neck ultrasonography for non-thyroid pathologies (lymphadenopathy, thyroglossal cyst, branchial cyst, etc.). Those with a history of thyroid disease were excluded from the study group.

### B mode ultrasound, color doppler, and SWE examinations

B mode, color Doppler and SWE imaging performed using a linear transducer probe (7.5–10 MHz) with a GE Logiq P9 medical system ultrasound machine (GE Healthcare, Chicago, IL, USA). To minimize confounding effects due to the operator variability, and because it is not possible to be blinded to the clinical appearance of cystinosis patients, all exams were performed by the same pediatric radiologist with the same scanner settings (B-mode, color gain, scale, PRF). All the scans were video recorded and assessed by the blinded second radiologist also. If the radiologists’ interpretations differed, consensus findings were used for a final decision. For the elastography results; the average of two radiologists’ measurements was presented.

Imaging of the thyroid gland was obtained in the supine position and the neck in mild extension. The thyroid was examined in transversal and longitudinal planes, and sonographic measurements of size were performed in transversal (two dimensions: width and depth) and longitudinal (one dimension: length) axes. The thyroid volume of each lobe calculated by the ellipsoid formula, and the total thyroid volume was calculated as the sum of both lobe volumes. Echo texture (normal-brighter than the surrounding muscles, hypoechoic- darker than the surrounding muscles), homogeneity (homogenous or heterogeneous), presence of septations, nodules, or any differences from normal thyroid gland appearance was noted.

On color Doppler the vascularity of both lobes was determined based on a visual scale previously reported by Schulz et al. (Table 1) [12].

**Table 1:** Color Doppler Classification, based on Schultz et al.’s study

Pattern 0	Parenchymal flow is absent
Pattern 1	Presence of mildly increased parenchymal flow
Pattern 2	Clearly increased color flow with a diffuse homogenous distribution
Pattern 3	Markedly increased color flow with a homogenous distribution

**Fig. 1:**
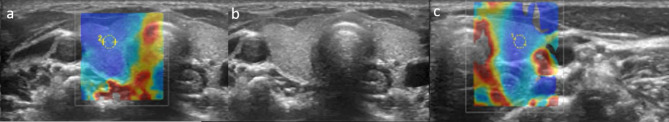
Quantitative evaluation of thyroid tissue with SWE of a patient number 7 (6-year-old girl). Transverse elastogram of right (**a**) and left (**c**) lobe; region of interest circle positioning was guided by the grayscale image (**b**).

Subsequently, real-time SWE was performed. SWE measurements were taken in the axial plane during normal breathing. In all participants, the left and right lobes were evaluated separately. Sampling for SWE was performed deeper than 0.5 cm, if possible, from parenchyma that did not include vessels. The region of interest was considered 3 × 3 mm. The quantitative evaluation of thyroid tissue stiffness was recorded in kPa. Averages of five measurements in each lobe were recorded (Fig. 1) for each readiologist.

### Ethics approval

This prospective study was conducted from December 2020 to June 2022 on patients with cystinosis and healthy controls. Informed written consent was obtained from all subjects’ legal guardians. The study was performed in accordance with the Ethics Guidelines of the Helsinki Declaration and was approved by the local Ethics Committee on 05.11.2020, approval number: 2020/22.

### Statistical analysis

Data were evaluated with the IBM SPSS Statistics 23 program. Descriptive statistics (mean, standard deviation, median, quartiles) were presented for numerical variables. The Spearman correlation was used for evaluation of correlation. The Mann–Whitney U test was used to compare the groups. P < 0.05 was considered for statistical significance.

## Results

### Patient characteristics

Sixteen paediatric patients (10 boys and 6 girls) diagnosed with cystinosis and thirty-four healthy children (16 boys and 18 girls) were included in the study. The mean ± SD age of cystinosis patients was 7.42 ± 3.15 (range: 0.98–12.23 years), while it was 9.01 ± 3.86 (range: 4.33–15.56 years) for healthy controls. There was no statistically significant difference between groups regarding age (p = 0.289).

The mean age at diagnosis for cystinosis was 13 months (range: 6–37 months). Eleven patients were diagnosed at or before 1 year of age, 3 patients were diagnosed between 1 and 2 years and 2 patients were diagnosed after 2 years of age. Two patients (cases 6 and 7) were siblings. All parents had parental consanguinity in the patient group. Eight patients (50%) were receiving levothyroxin treatment during the study for hypothyroidism while none of the control group was receiving any thyroid treatment. The initiation and maintenance doses of levothyroxine and follow-up of the treatment in cystinosis patients with hypothyroidism were adjusted according to the guidelines [13].

Patient characteristics (demographic, clinical and laboratory parameters) of the cystinosis group were presented in Table 2.

**Table 2:** Demographic, clinical and laboratory parameters of the cystinosis patients group

Patient no	Gender	Age (decimals)	Height (cm)	Weight (kg)	BMI	Age at the diagnosis (months)	eGFR	CKD Stage	Thyroid function	Levothyroxine treatment
**1**	M	4,49	97	15	15,9	6	106	G1	Hypothyroidism*	*Present*
**2**	M	9,99	121	21,9	15	24	73	G2	Normal	None
**3**	F	0,98	66	6	13,7	6	157	G1	Hypothyroidism*	*Present*
**4**	M	11,81	113	20	15,6	10	29	G4	Normal	None
**5**	F	5,63	89	15	18,9	6	224	G1	Hypothyroidism*	*Present*
**6**	M	5,72	108	16	13,7	8	165	G1	Normal	None
**7**	M	6,83	110	17,5	14,4	11	168	G1	Normal	None
**8**	F	5,23	96	14	15,2	9	138	G1	Hypothyroidism*	*Present*
**9**	F	6,13	96	15	16,3	6	107	G1	Normal	None
**10**	F	11,1	119	22,5	15,9	12	40	G3b	Normal	None
**11**	M	5,68	90	11,2	15	13	110	G1	Normal	None
**12**	M	10,94	110	24	19,8	37	*9*	*G5*	Hypothyroidism*	*Present*
**13**	F	9,1	118	20,7	14,8	16	60	G2	Hypothyroidism*	*Present*
**14**	M	12,23	120	25	17,3	30	35	G3b	Hypothyroidism*	*Present*
**15**	M	7,75	112	16,2	12,9	9	*10*	*G5*	Hypothyroidism*	*Present*
**16**	M	5,1	89	13	16,4	7	140	G1	Normal	None

eGFR: estimated Glomerular Filtration Rate; BMI: body mass index; CKD Stage: Chronic Kidney Disease Stage;

* The cases reported as hypothyroidism were euthyroid at the time of the study as they were receiving levothyroxine treatment

## B mode ultrasound and color doppler evaluation

B Mode ultrasound imaging revealed lower echogenicity and heterogeneous echo texture in 7 out of 16 (44%) cystinosis patients. Two patients exhibited extremely heterogeneous and small thyroid tissue, while 7 patients had normal ultrasound findings within the expected range (Table 3). The control group showed normal imaging patterns in thyroid ultrasound. The abnormal sonographic patterns observed in cystinosis patients were independent of their current age, age at diagnosis, or thyroid hormone levels.

**Table 3:** Ultrasound, Color Doppler and SWE findings of cystinosis patients group

Patient number	B-Mode ultrasound findings	Total thyroid volume(ml)	Standard deviation of thyroid volume	Color Doppler pattern	Mean Right lobe kPa	Mean Left lobe kPa	Mean Total kPa
**1**	Normal	1,69	0,19	0	3,36	5,7	4,53
**2**	Normal	4,2	0,35	0	5,7	5,9	5,8
**3**	Normal	0,49	-1	0	5,73	7,2	6,465
**4**	Hypoechoic, Heterogeneous	2,86	0,23	1	6,5	6,9	6,7
**5**	Normal	3,54	0,92	1	4,9	6,04	5,47
**6**	Hypoechoic, Heterogeneous	2,6	-0,03	2	4,2	3,5	3,85
**7**	Normal	1,59	-1,05	1	4,1	7,4	5,75
**8**	Normal	2,05	-0,59	0	3,8	7,5	5,65
**9**	Hypoechoic, with small echogenic focuses	1,08	-1,57	1	7,7	8,5	8,1
**10**	Hypoechoic, with small echogenic focuses	2,35	-1,56	0	8,9	7,2	8,05
**11**	Hypoechoic, heterogeneous	0,91	-1,74	2	6,7	10,2	8,45
**12**	Small atrophic, heterogeneous	0,22	**-2,39**	0	non diagnostic	non diagnostic	non diagnostic
**13**	Hypoechoic, heterogeneous	2,35	-0,92	2	3,9	4,43	4,165
**14**	Small atrophic, heterogeneous	0,6	**-2,68**	0	non diagnostic	non diagnostic	non diagnostic
**15**	Normal	2,02	-0,62	0	3,59	4,6	4,1
**16**	Hypoechoic, heterogeneous	1,57	-1,07	2	6,5	6,2	6,35

The total thyroid volume ± SD of cystinosis patients was 1.88 ± 1.1 ml (range: 0.22–3.54 ml) (Table 3), whereas the healthy control group had a total thyroid volume ± SD of 3.41 ± 1.89 ml (range: 1.07–8.67 ml). There was no significant correlation between age and total thyroid gland volume (p = 0.560) in cystinosis patients. However, a positive correlation was found between age and total thyroid gland volume in the control group (p = 0.000). The total thyroid volume of cystinosis patients was lower than that of the healthy control group (p = 0.005) (Table 4).

**Table 4:** Evaluation of the total sonographic thyroid volume (ml) and thyroid tissue stiffness (mean total kPa) of cystinosis patients and healthy control group

	Cystinosis Patients			Controls			Total			p
	Mean	SD	Median	Mean	SD	Median	Mean	SD	Median	
**Age**	7,42	± 3,15	6,48	9,01	± 3,86	8,09	8,50	± 3,69	7,66	0,289
**Total sonographic thyroid volume (ml)**	1,88	± 1,10	1,86	3,41	± 1,89	2,89	2,92	± 1,81	2,47	0,005*
**Mean Total kPa of Thyroid Gland**	5,96	± 1,51	5,78	8,70	± 3,76	7,48	7,90	± 3,49	7,01	0,003*

* P value < 0.05 is considered significant

**Fig. 2:**
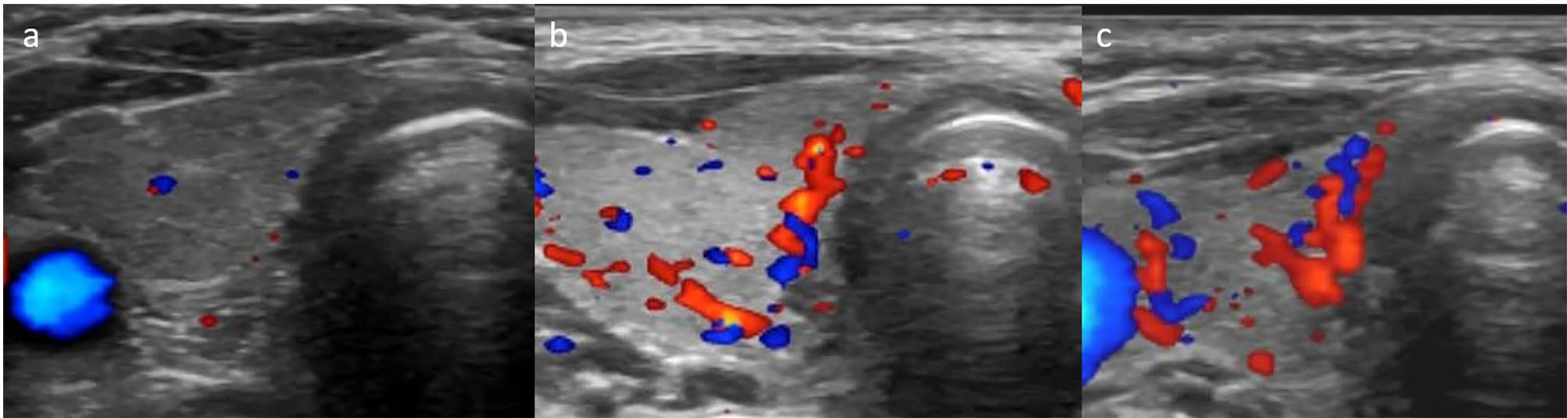
Examples of color Doppler ultrasonography patterns of patients; (**a**) patient number 10-pattern 0; (**b**) patient number 7-pattern 1; patient number 11-pattern 2

Among the cystinosis patients, 8 (50%) had a color Doppler pattern classified as normal (pattern 0). Increased vascularization was observed in 8 patients (50%), with 4 patients showing pattern I and 4 patients showing pattern II (Fig. 2). None of the patients exhibited pattern III. The color Doppler findings in the control group were all normal, consistent with pattern 0.

### SWE evaluation

Two cystinosis patients were excluded from the elastographic evaluation due to extreme thyroid tissue atrophy (below − 2 SD of normal ranges), making precise elastographic measurements impossible. The mean ± SD of SWE tissue stiffness measurements of the total thyroid parenchyma of cystinosis patients was 5.96 ± 1.51 kPa (range: 3.85–8.45 kPa). There was no significant correlation between current age, age at diagnosis, thyroid hormone level status, and kPa values. On the other hand, the mean ± SD of SWE measurements in the healthy control group’s total thyroid parenchyma were 8.7 ± 3.76 kPa (range: 4.1–23 kPa). The average kPa values of cystinosis patients were lower than those of the healthy control group (p = 0.003) (Table 4).

## Discussion

**Fig. 3:**
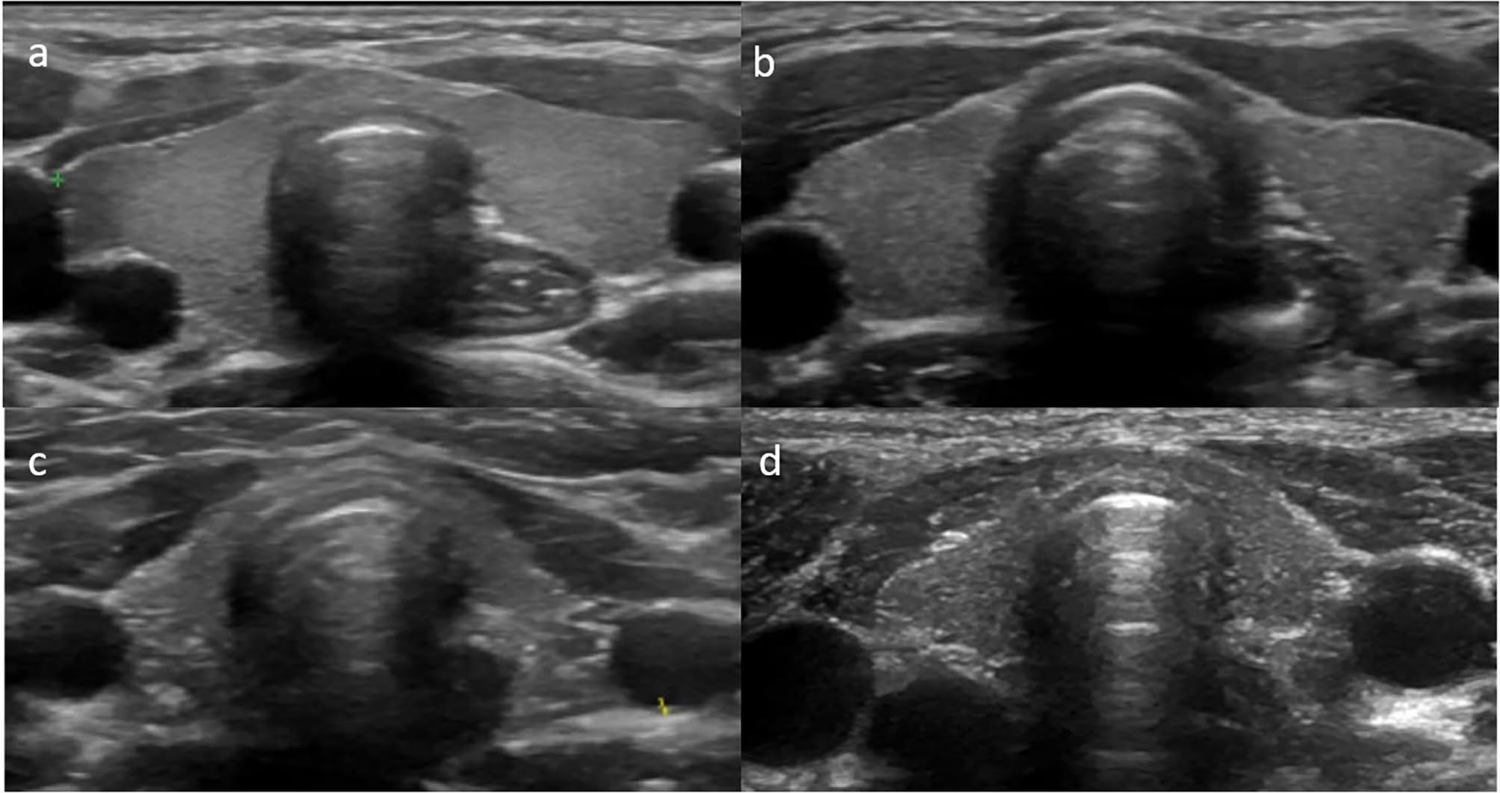
Transverse USG images of thyroid tissue of healthy control and patients. (**a**) healthy 9-year-old girl, USG image shows the homogeneous echogenicity and uniform texture of the normal thyroid tissue, the sizes of the gland are within normal limits; (**b**) patient number 4 (11-year-old boy), USG image shows slightly lower echogenicity and minimally heterogeneous echotexture, gland sizes are within normal limits; (**c**) USG images of patient number 12 (10-year-old boy) and patient number 14 (**d**) (12-year-old girl) both show excessively atrophic and very heterogeneous thyroid tissue

**Fig. 4:**
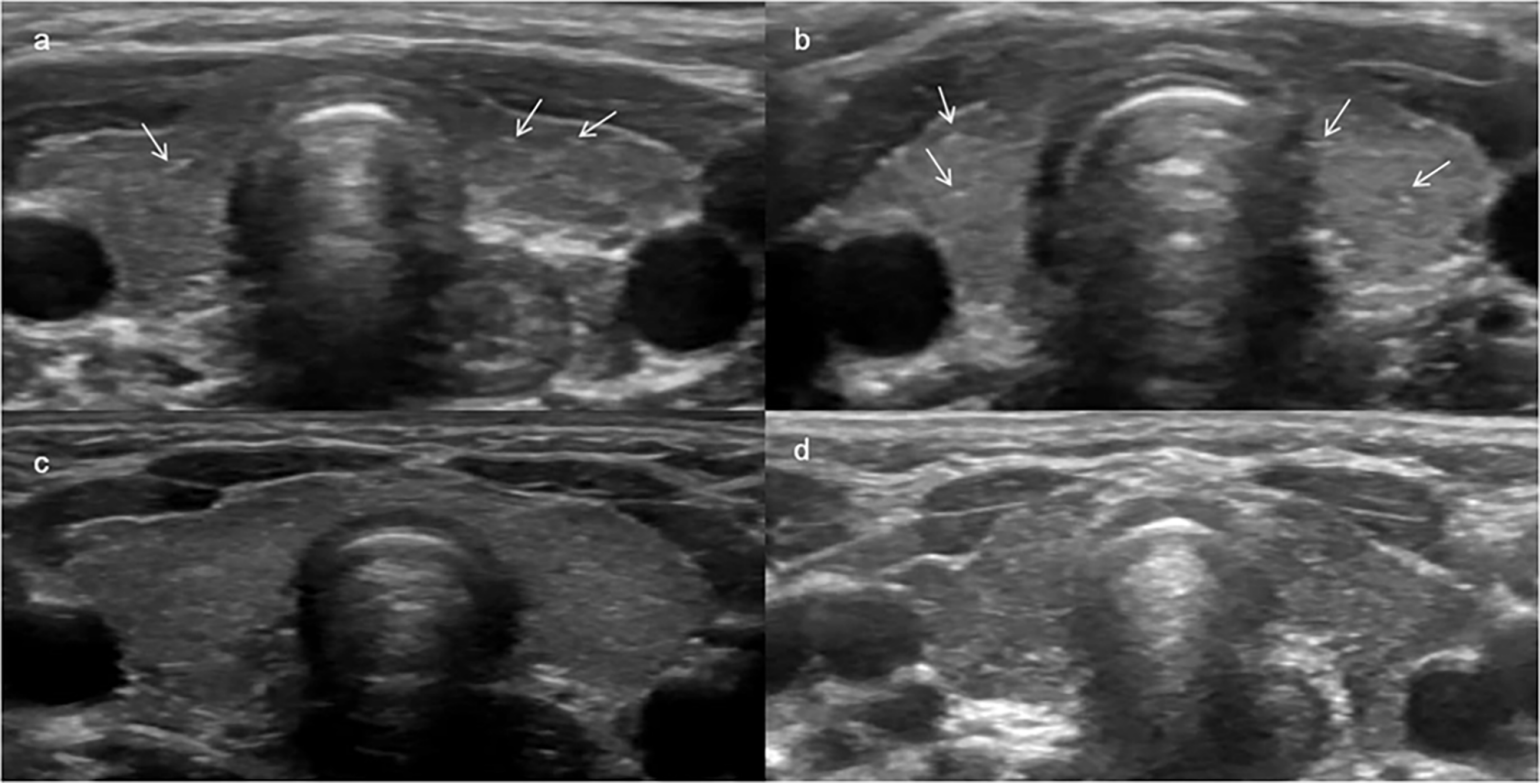
Transverse USG images of thyroid tissue of patients. (**a**) patient number 9 (6-year-old girl), USG image shows homogeneous echotexture and small hyperechoic spots (arrows); (**b**) patient number 10 (12-year-old girl), USG image shows homogeneous echotexture and a slight increase in echogenic septations and small hyperechoic spots (arrows’ heads); (**c**) patient number 11 (6-year-old boy), USG image shows lower echogenicity and slightly heterogeneous echotexture; (**d**) patient number 16 (5-year-old boy), USG image shows lower echogenicity and diffuse heterogeneous echotexture, note slightly lobulated contours

To the best of our knowledge, this is the first study evaluating thyroid gland B mode ultrasonography, color Doppler and SWE findings in cystinosis patients. Ultrasound imaging revealed lower echogenicity and heterogeneous echo texture in 7 of the 16 cystinosis patients (Figs. 3 and 4). On SWE, the thyroid tissue stiffness was established to be lower than that of the controls.

In our study, the most common pathologic appearance on thyroid sonography was a diffusely altered and hypoechoic parenchyma, which has also been reported in many other previously known diffuse infiltrating diseases. In Hashimoto thyroiditis, which is a well-known prototype of diffuse infiltrating diseases involving thyroid tissue, lower echogenicity is one of the diagnostic sonographic findings and is a result of a lymphocyte infiltration process [14]. In Fabry disease, which is a more frequent lysosomal disease than cystinosis, Faggiano et al. [15] reported that the thyroid US pattern was mildly hypoechoic in most of the patients in their study. Therefore, we can rationally assume that even under cysteamine treatment, because of the low cellular proliferation rate (total of six to seven mitoses during a lifespan [16]) of thyrocytes, the decreased sonographic echogenicity in cystinosis patients might be due to the cystine crystal accumulation and/or disease infiltration process. The reported abnormal thyroid US pattern was independent of current age, age at diagnosis or beginning of cysteamine treatment. The same findings regarding B mode ultrasound were also established in Fabry disease patients [15].

In two patients (patients 9 and 10), while the normal echogenicity and homogeneity of the thyroid gland were preserved, a slight increase in echogenic septations and small hyperechoic spots were observed. These findings may contribute to early stages of concomitant thyroiditis, fibrosis or even different presentations of thyroid tissue involvement of cystinosis because of differences in genotypic mutations of the CTNS gene. The wide spectrum of mutations of the CTNS gene [3] might also be the source of different sonographic presentations.

The thyroid gland sizes and total thyroid volume of cystinosis patients were lower than in the healthy control group (p = 0.005). Furthermore, while the thyroid gland sizes and volumes positively correlated with the age in the control group, there was no correlation in the cystinosis patients. The thyroid gland volumes of most of the patients still fell within the relatively wide range considered normal for children [17] except for two patients. These patients were diagnosed and had begun treatment somewhat later than the others (30 and 37 months), and their thyroid glands were excessively atrophic (below − 2.5SD). These findings support previous reports that early treatment with cysteamine slows endocrinologic deterioration [9, 14, 18–20].

Our second important finding was that the mean SWE value, representing the stiffness of thyroid tissue, was lower (5.96 kPa) in cystinosis patients compared to the healthy group (8.70 kPa) (p < 0.003). Even in various diseases such as chronic thyroiditis, malignant thyroid nodules, and amyloid deposition, an increase in thyroid tissue stiffness is typically attributed to fibrosis or inflammation we did not find any studies reporting lower thyroid gland elastography values in any disease. Therefore, we hypothesize that this finding can be explained by evaluating tissue dynamics that affect tissue stiffness. Guimarães et al. have stated that tissue stiffness is primarily determined by fundamental components such as cells and the extracellular matrix and also cell-cell and cell-matrix interactions and adhesions [21–23]. They further claim that increased tissue stiffness is mainly dependent on a desmoplastic deposition and crosslinking of extracellular matrix fibers, as well as fibroblast proliferation and differentiation into myofibroblasts. Conversely, the loosening of extracellular matrix fibers and adhesions between cells may be responsible for the decrease in tissue stiffness. Therefore, the decrease in tissue stiffness in cystinosis patients might be associated with the conditions mentioned above. On the other hand, a unique study reported 40 years ago revealed histopathological findings of the thyroid gland in cystinosis patients, indicating a gradual atrophy of the follicular tissue without any evidence of new fibrous tissue proliferation during the progression of cystinosis [24]. Considering the aforementioned tissue dynamics, conditions influencing tissue stiffness, and previously reported thyroid gland atrophy in untreated cystinosis patients, we propose that the lower tissue stiffness in cystinosis might be attributed to alterations in cell-cell or cell-extracellular matrix interactions, primarily due to cell apoptosis. Furthermore, a recent mice model study suggested a complex pathogenesis of thyroid dysfunction in cystinosis, including accelerated thyrocyte turnover, increased cell proliferation, enhanced apoptosis linked to endoplasmic reticulum stress, impaired thyroglobulin production, and altered endolysosomal trafficking and iodothyroglobulin processing [25], further emphasizing apoptosis as a contributing factor to thyroid dysfunction in cystinosis, which supports our hypothesis. Although cell proliferation and thyrocyte turnover mentioned in the study are important factors affecting tissue stiffness alterations, we believe that enhanced apoptosis has the most significant impact on elastography values.

The other important finding of our study was the increase in the vascularity of thyroid gland in the half of the patients.Although this specific finding has not been previously reported, a recent study by Besouw et al. highlighted that cysteamine concentrations similar to those found in the plasma of individuals with cystinosis can stimulate the proliferation of human dermal microvascular endothelial cells and may lead to angioendotheliomatosis triggered by cysteamine [26]. Therefore, vascular proliferation due to cysteamine might be the other important cause of the increase in vascularity and decrease in tissue elastography stiffness. We think that this important finding needs to be evaluated in larger patient groups. Our study has several limitations, including its relatively small sample size and descriptive nature. But since our study group consisted of pediatric patients with very rare disease; this limitation may be considered natural. Another limitation may be the lack of leukocyte cystine levels, which are crucial in detecting adherence in cysteamine treatment, altering clinical stage and probably radiological imaging findings. However, the emphasis of this study was on reporting the ultrasound and elastography findings in cystinosis rather than evaluating adherence or effectiveness of treatment. A third limitation may be the fact that the first radiologist was not blinded to the patients’ clinical conditions, which might have led to bias in terms of qualitative analysis of thyroid gland sonographic appearance, but we assume to have partially overcome this limitation by including a second reviewer, blinded to patients’ clinical conditions. The other limitation of our study is that we excluded two patients from the study’s elastographic evaluation part, because of the extremely atrophic (below − 2 SD of normal ranges) thyroid tissue, making elastographic measurements impossible. We may assume that the SWE values of their end-stage organs could be increased. Therefore, excluding these patients may bias our SWE analysis. But unfortunately, SWE measurements of these patients’ thyroid tissue, were impossible. Due to the small size of our cystinosis patient group and the lack of age-specific SWE elastography nomograms, we could not evaluate patients SWE results separately based on age groups, which may be also another limitation. The final limitation to consider is that the stiffness measurements were performed in transverse orientation only; the longitudinal measurements were not performed. However, it was previously reported [27] that although the longitudinal orientation was somewhat favoured, there is flexibility for the imager to use both orientations for SWE measurements, and this possible limitation in this study was mitigated by acquiring multiple measurements from each lobe.

## Conclusion

This is the first study evaluating thyroid gland B mode ultrasonography, color Doppler and SWE findings in cystinosis patients. Ultrasound imaging revealed lower echogenicity and heterogeneous echo texture in nearly half of the cystinosis patients unrelated to current age, age at diagnosis or thyroid hormone level status. Even though the thyroid gland volumes of most of the patients fell within the relatively wide range quoted for healthy children, thyroid gland volumes were lower in cystinosis patients even under cysteamine treatment. Also increase in vascularity of the gland and decrease in tissue stiffness may be attributed to the hypothesis that cysteamine treatment still cannot completely prevent the disease infiltration process, even underscores the cystine accumulation and decelerates the progression to thyroid dysfunction.

## Data Availability

All data supporting the findings of this study are available within the paper. Also at https://docs.google.com/document/d/1gYMBEOri-IuiwpTf2A2kppsMkZTiX7w1/edit?usp=sharing&ouid=112455242974227852003&rtpof=true&sd=true.
